# Cancer, Senescence, and Aging: Translation from Basic Research to Clinics

**DOI:** 10.4061/2011/692301

**Published:** 2011-10-20

**Authors:** Matilde E. LLeonart, Amancio Carnero, Rosanna Paciucci, Zhao-Qi Wang, Noam Shomron

**Affiliations:** ^1^Oncología y Patología Grupo, Vall d'Hebron Institut de Recerca, 08035 Barcelona, Spain; ^2^Unitat de Recerca Biomédica, Instituto de Biomedicina de Sevilla, 41013 Sevilla, Spain; ^3^Genomic Stability Laboratory, Leibniz Institute for Age Research, 07745 Jena, Germany; ^4^Genome High-Throughput Sequencing Laboratory, Department of Cell and Developmental Biology, Sackler Faculty of Medicine, Tel Aviv University, Tel Aviv, Israel

Unravelling the molecular basis of malignancy is a challenging process of great priority, as cancer rates are increasing worldwide and because certain cancer types are still incurable. The urgent need for novel treatment modalities based upon recent discoveries at the genetic and epigenetic level necessitates a strong collaboration between researchers and clinicians to work toward a common aim: the control of the carcinogenic process in order to ultimately achieve a 100% cure rate.

In the last 20 years, a myriad of discoveries at the molecular level have been accomplished, especially with the completion of the “Human Genome Project.” This special issue focuses on how senescence affects tumourigenesis and how novel senescence-related therapeutic approaches could be used to benefit tumour regression and eradication efforts.

Mammalian cells have developed complex defence mechanisms, such as apoptosis, growth or cell cycle arrest, and senescence, to combat uncontrolled proliferation caused by external stimuli (e.g., carcinogenic agents). Replicative senescence occurs when somatic cells spontaneously decline their growth rate in continuous culture due to an increasing number of population doublings, eventually terminating in a quiescent but viable state. Importantly, senescence has been observed in patients with premalignant tumours but has not been detected in malignant tumours. Clear evidence points to a crucial role of cellular senescence in counteracting malignant transformation. Therefore, in order to eradicate cancer, key molecules (proteins, microRNAs, etc…) and processes important in senescence could be targets for therapeutic intervention.

The papers collected in this issue deal with the above-mentioned key senescence factors.

1. All important oncogenes and tumour suppressor genes crucial in this process are shown. How and to what extent their targeting would be effective in cancer therapeutics is reviewed.
2. Recently, a specific group of proteins called Sirtuins, which are part of an evolutionarily conserved family of NAD-dependent protein deacetylases/ADP-ribosyltransferases, have been identified as key components in senescence and aging. The relationship of Sirtuins with genomic instability and their influence on telomerase and tumourigenesis is discussed.
3. DNA-damage response and its link with self-renewal and senescence are reviewed as determinant processes in which senescent cells may decide how their genetic backgrounds and protein statuses can promote or prevent carcinogenesis.
4. MicroRNAs, short noncoding RNA molecules of ~22 nucleotides, are key post-transcriptional regulators of gene expression. MicroRNAs have an important role in tumour development, progression, chemosensitivity and cellular senescence. Decoding microRNA function is required for the development of novel therapies, such as restoring tumour suppressor-microRNAs and targeting onco-microRNAs with anti-miR technology. All of these approaches are extensively reviewed.

Moreover, abundant evidence suggests that senescence plays an important role in aging. The paradoxical role of senescence as a protective mechanism against the eradication of cancer might be detrimental to the possible contribution of senescence to aging. The molecular regulation of senescence in cancer and aging is discussed.

Overall, the potential of cellular senescence to be used as a target for anticancer therapy is a close reality in the clinical practice. In this issue, therapeutic strategies are fully considered, and their applications in each case are proposed.

*Matilde E. LLeonart* *Matilde E. LLeonart* *Amancio Carnero* *Amancio Carnero* *Rosanna Paciucci* *Rosanna Paciucci* *Zhao-Qi Wang* *Zhao-Qi Wang* *Noam Shomron* *Noam Shomron*

